# Analysis of Nematode Motion Using an Improved Light-Scatter Based System

**DOI:** 10.1371/journal.pntd.0003523

**Published:** 2015-02-19

**Authors:** Chuck S. Nutting, Rob R. Eversole, Kevin Blair, Sabine Specht, Thomas B. Nutman, Amy D. Klion, Samuel Wanji, Michel Boussinesq, Charles D. Mackenzie

**Affiliations:** 1 Department of Biological Sciences, Western Michigan University, Kalamazoo, Michigan, United States of America; 2 Department of Chemistry, Western Michigan University, Kalamazoo, Michigan, United States of America; 3 Institute for Medical Microbiology, University Hospital Bonn, Bonn, Germany; 4 Laboratory of Parasitic Diseases, NIH, Bethesda, Maryland, United States of America; 5 Institut de Recherche pour le Développement, Montpellier, France; 6 Research Foundation in Tropical Diseases and Environment, University of Buea, Buea, Cameroon; 7 Department of Pathobiology and Diagnostic Investigation, Michigan State University, East Lansing, Michigan, United States of America; University of California, San Francisco, UNITED STATES

## Abstract

**Background:**

The detailed assessment of nematode activity and viability still remains a relatively undeveloped area of biological and medical research. Computer-based approaches to assessing the motility of larger nematode stages have been developed, yet these lack the capability to detect and analyze the more subtle and important characteristics of the motion of nematodes. There is currently a need to improved methods of assessing the viability and health of parasitic worms.

**Methods:**

We describe here a system that converts the motion of nematodes through a light-scattering system into an electrical waveform, and allows for reproducible, and wholly non-subjective, assessment of alterations in motion, as well as estimation of the number of nematode worms of different forms and sizes. Here we have used *Brugia sp.* microfilariae (L1), infective larvae (L3) and adults, together with the free-living nematode *Caenorhabditis elegans*.

**Results:**

The motion of worms in a small (200ul) volume can be detected, with the presence of immotile worms not interfering with the readings at practical levels (up to at least 500 L1 /200ul). Alterations in the frequency of parasite movement following the application of the anti-parasitic drugs, (chloroquine and imatinib); the anti-filarial effect of the latter agent is the first demonstrated here for the first time. This system can also be used to estimate the number of parasites, and shortens the time required to estimate parasites numbers, and eliminates the need for microscopes and trained technicians to provide an estimate of microfilarial sample sizes up to 1000 parasites/ml. Alterations in the form of motion of the worms can also be depicted.

**Conclusions:**

This new instrument, named a "WiggleTron", offers exciting opportunities to further study nematode biology and to aid drug discovery, as well as contributing to a rapid estimate of parasite numbers in various biological samples.

## Author Summary

Assessment of the health and number of nematodes still relies heavily on subjective monitoring of their motion. Although less-subjective techniques exist that utilize the motility as the primary indicator, the current approaches tend to be designed for use with larger worms and not for early developmental stages. We have describe here a sensitive technique that converts the motion of nematodes into electrical waveforms, which then be used for an estimation of the number parasites present, and for detailed analysis of alterations in their movements. Using parasites of different sizes, including different stages of *Brugia sp*. and *Caenorhabditis elegans*, we have shown that the system can analyse samples containing up to 1000 microfilariae/ml, and can be used to used to detect the decrease in motility as a worm loses viability. We have also demonstrated its use in assessing the effects of chloroquine and imatinib on filariae. This sensitive technique is likely to be value to research and field laboratories where there is a need to rapidly estimate the number of parasites present in liquid samples, and can be used in drug screening programs to assess the effects of different anthelminthics.

## Introduction

Understanding nematodes, the most numerous multicellular organisms known, is important for the advancement of both basic biological knowledge and improving global health. The free-living nematode *Caenorhabditis elegans*, for example, has been a model for investigating the fundamentals of genetics and aging. In the medical and veterinary world parasitic nematodes cause of some of the most debilitating and serious diseases that contribute to the global challenges in achieving improved human and animal health. Intestinal and vessel-dwelling nematodes infect more than 2 billion people with serious health and economic consequences [1], Much of the laboratory based research into parasitic nematodes to date has utilized either relatively subjective *in vitro* assays based on observer interpretation, or have been focused on *in vivo* approaches that often still involve subjective approaches.

There have been a number of non-subjective mechanical techniques developed to study nematode activity, including those that measure changes in motility. In 1986 a micromotility meter, a comparatively inexpensive and easily operated device, was described for the quantification of motility of large nematodes [2]. This, and more recent devices, such as the real time cell monitoring system based on impedance [3], have been used to investigate the effects of anthelminthic agents on parasitic nematodes. The screening of compounds for potential new anti-parasite candidates has increased in recent years as global health programs focus more sharply on eliminating the major neglected tropical infections caused by helminths including nematodes such as *Onchocerca volvulus, Wuchereria bancrofti and Brugia sp*. [4]. This push to find new drugs that will work against these parasites requires improved technologies including better use of the commonly used parameter of parasite motion as a predictor of parasite health [5–8]. Present techniques are lacking in utility; some address only the larger stages of parasites and cannot quantify or measure microscopic larval stages [5], whilst others are not compatible with use in remote field settings because of cost or complexity [6–8].

*C. elegans* has an episodic swimming pattern that has been well-documented [9], and the motion of nematodes has commonly been used as an indicator of the health of the worms. Filarial parasites in particular have a very specific pattern of motion, such as the “filarial dance” seen in lymphatic filariasis using ultrasonography [10]; this motility is reduced after administration of anthelmintic treatment.

Here a technology is described that improves the capture the details of the complex motion of these parasites in solution and the characteristics of alterations in this motion. This system also allows evaluation of potential anti-worm agents in vitro by quantifying the number of viable worms present in test samples. To describe our new system we have primarily used *Brugia pahangi*, a filarial worm similar to the 3 species that cause human lymphatic filariasis, and have also included data on *C. elegan*s, the archetypical nematode.

## Materials and Methods

### Ethics statement

The animals used to supply the parasites used in this study were infected and maintained under standard institutional approval monitored and proved by the Animal Use Committee of Western Michigan University (WMU). Animals were maintained in the approved and monitored animal facilities at WMU.

### Motion sensing equipment

**Fig 1:**
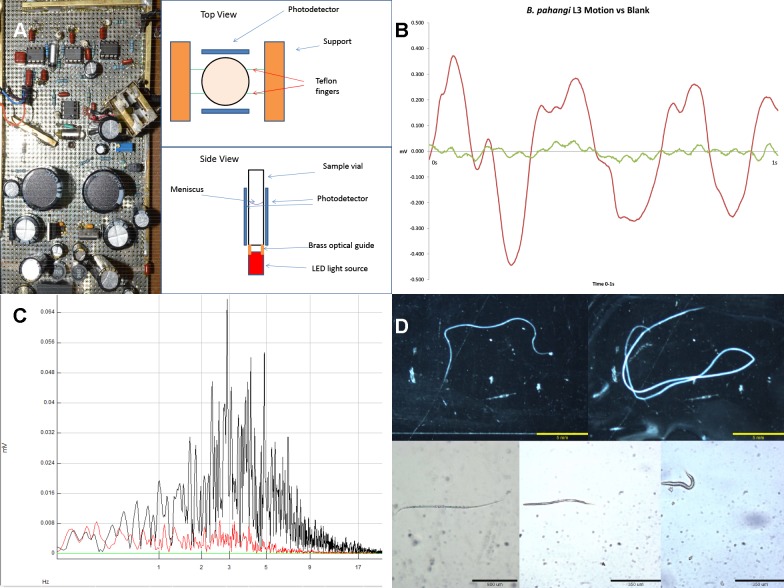
a. Inside of the Wiggle-tron with top and side view of sample mount and collection apparatus. **b**. One second tracing of a single *Brugia pahangi* L3 motion vs blank. **c**. FFT of 10 healthy (black) and 10 degraded (red) L3’s overlaid. **d**. Images of *Brugia pahangi* adult male, bar 5000um (1); female, bar 5000um (2); L3, bar 800um (3); and microfilaria, bar 350um (4); as well as an image of *Caenorhabditis elegans* L1, bar 350 um (5).

The WiggleTron (WT) (**Fig. 1A**) operates on many of the same principles as its predecessor, the B&P Instruments Micromotility Meter [2]. The WT has, however, improved sensitivity in detection, decreased noise, better placement of the photo-detectors, and increased sampling rate as compared to the B&P machine. The WT ultimately converts sample organism movements into an analog voltage signal which once sampled is analyzed for its amplitude and frequency composition time. The sample-containing vial is illuminated from below by a white LED emitting ~5 milliwatts of visible optical power into the base of the vial. The LED and sample tube are separated by an opaque ring which retains stray light, reduces thermal conduction into the sample vial, and prevents damage to the LED lens. Temperature within the sample vial is consistently 32 ± 1°C. The light passes up through the sample reflecting dynamically off any movement within the vial, onto large PIN photo-detectors, connected in parallel, flanking the sides of the vial. Unlike the original micro-motility meter, these photo- detectors span vertically from the base of the vial up to just below the meniscus level of the liquid sample. This reflected light energy is converted to an analog of the movement, amplified at 0.40, 4.0 or 40 μV/pA to accommodate various sizes of sample organism, using negative feedback resistance to determine the optimal situation. The sample vial is held firmly by support fingers that in turn are stabilized by a superstructure that also supports and aligns the photo-detectors. All samples were evaluated in 31mmH X 6 mm OD borosilicate glass tubes (Sigma, pn.24715) with 200μl samples.

This system could be miniaturized and made suitable for use in field situations, as well as be constructed to test multiple samples at the same time allowing for large scale screening of compounds. The cost of the basic equipment and software components for a simple version of this system was approximately $4,500–$6000.

### Measurement and analysis

Tubes were placed within the WT over the light source and allowed to equilibrate for at least one minute prior to initiating acquisition. Each sample population (containing parasites) as well as control (medium only) was prepared in triplicate. The analog voltage output signal was digitized at 1 kHz with a DT9816 (Data Translation, Inc., Marlboro, Massachusetts, USA) 16 bit ADC controlled by Quick-DAQ software (Data Translation, Inc., Marlboro, Massachusetts, USA). The recording duration used for pilot studies was 30 seconds, however after experimentation it was found that to accommodate the 2^N^ time requirement for low frequency FFT resolution of 0.1Hz [11], this testing time be standardized for all samples at 33 seconds. The proprietary Quick-DAQ *.hpf data files were converted to *.csv files containing time interval and voltage amplitude. The voltage amplitude was multiplied by 3276 then converted to RMS value to represent better the sample’s bit depth for 16 bit resolution of the ±10 V input range of the DT 9816, making the resulting amplitude values integer numbers and thus easier to interpret and compare quickly. This number (3276) was selected as it is the factor that best converts bit number into an arbitrary unit that allows for easier comparisons; this technique is commonly used in electronic science [12]. Thus it is not a specific “unit” as such but a non-dimensional unit used solely for the comparison between two or more samples studied by this equipment.

Each sample was run 3 times on the WT. The mean and standard deviation for each sample was calculated using the scaled RMS values from these individual runs. This yields a number that can be used to compare the motion of the worms in each sample based on the amplitude of motion. Dead parasites (transferred to 95% ethanol), then transferred back to fresh medium followed the same procedure for sample preparation, measurement, and analysis. Further, the (*.csv) files can be imported into SignalLab SIGVIEW (Mitov Software LLC, Moorpark, California, USA). This software allows FFT analysis to look at differences in the frequency of motion of the sample and highlight differences graphically at different frequencies of movement.

### Parasite material

***Brugia pahangi* microfilariae**. *B. pahangi* microfilariae were obtained from Filariasis Research Reagent Resource Center (FR3, Atlanta, Georgia, USA). Upon receipt, microfilariae were placed in approximately 25ml of fresh medium (RPMI 1640 supplemented with 10% fetal bovine serum (FBS) and 1% penicillin and streptomycin) and agitated till at a uniform distribution within the medium. 10 ul of this suspension was then transferred to 100 ul of fresh medium in a well on a 96-well plate. This was repeated 4 times. The contents of the wells were examined under light microscope to determine parasite health (highly active and with normal anatomical appearance). Once parasites were determined to be healthy and free from debris, all 4 wells were counted for living parasites to determine the density of microfilariae in suspension. This density number was then used to generate dilutions to allow us to prepare samples ranging from 10 to 50,000 worms suspended in 200 ul of medium. Each sample was then run on the WT and analyzed as described earlier the results from each population were averaged to provide a snapshot of motility amplitude of the range of populations. Samples were then prepared using dead microfilariae from 10–50,000 worms and run and analyzed in the same manner, populations of live versus dead worms could then be compared.

Since the samples were prepared in triplicate each population could then be averaged to generate a plot of population as a function of measured and analyzed RMS amplitude. For tube populations from 0–1,500 worms, this plot yielded a function capable of predicting the population of parasites within the sample tube. Unknown population samples were then prepared, run, and analyzed as described above. The equation resulting from the known population was used to calculate the population of microfilariae in the unknown tubes. The population of each of the unknown tubes was then found using the same method used to find concentrations of microfilariae described previously and compared to the calculated population.

***Brugia pahangi* infective larvae**. *B. pahangi* infective larvae (L3’s) were obtained from FR3. Upon receipt, L3’s were placed in approximately 1ml of fresh medium (RPMI 1640 supplemented with 10% fetal bovine serum (FBS) and penicillin and streptomycin) and agitated to mix uniformly within the medium. 20ul of this suspension was then transferred to a small petri dish containing approximately 2ml of medium. The dish was investigated under light microscopy to determine parasite health. Once parasites were determined to be healthy and free of debris, they were counted to determine the density of L3’s in suspension. Samples containing between 10 and 200 L3’s in 200 ul of medium were then prepared. Samples with less than 10 worms were also prepared by pipetting a single worm at a time under the stereoscope into the same type of vials described previously; each sample prepared in this manner also had a final volume of 200 ul. Samples were prepared in triplicate. All samples were then covered with Parafilm (Pechiney Plastic Packaging Co., Chicago, USA) and placed in the freezer for 2 days after live analysis. Afterward, the tubes were thawed and inspected to make sure the parasites were dead. Tubes with the dead parasites were then run on the WT and analyzed as previously described. Both live and dead L3’s were then compared at each worm population level.

***Brugia pahangi* adult parasites**. Adult *B. pahangi*, both male and female were obtained from FR3. Upon receipt, parasites were placed in approximately 5 ml of fresh medium (RPMI 1640 supplemented with 10% FBS and penicillin and streptomycin) in a large petri dish. Individual worms were picked up using a glass Pasteur pipette and transferred into 200 ul of medium in the same type of vials described previously, 1 parasite per vial. Samples were run on the WT and analyzed as previously described. These samples were then covered with Parafilm and placed in the freezer for 2 days. Afterward, the tubes were thawed and inspected to make sure the parasites were dead. Tubes with the dead parasites were then run on the WT and analyzed as previously described and their results compared to living adult worms.

***Caenorhabditis elegans* L1 stage**. Mature, egg-producing *C. elegans* were bleached. The eggs were harvested, washed and allowed to hatch on a plate containing agar with no food, arresting their development at the L1 stage to ensure a uniform larval stage for analysis. Samples from 0–100 worms were prepared as previously described, however rather than using medium *C. elegans* were run in normal saline (0.9% NaCl) to ensure that they remained arrested in L1. Samples of live and dead *C. elegans* were run as previously described, FFT frequency analysis was done as previously described, using 100 L1’s.

### Drug treatment effects on motility

**Infective larvae (L3)**. Twenty-one samples, each containing two *B. pahangi* L3’s were prepared in 198 ul of medium in the same vials described earlier. These 21 samples were grouped into 7 groups of three. These groups included one control group, and three treatment groups for the drugs imatinib mesylate (Gleevec, Novartis) and chloroquine (Sigma-Aldrich Co, New Jersey, USA), both at levels of 100nM, 500nM, and 100uM. Each sample was run prior to treatment on the WT. Two microliters of medium were added to each tube in the control group, two microliters of a 10uM drug solution were added to the tubes in the 100nM groups, two microliters of a 50uM drug solution added to the tubes in the 500nM groups, and two microliters of a 10mM drug solution were added to the 100uM groups. Each tube was covered with Parafilm M Barrier Film (Bemis NA, Neenah, Wisconsin, USA) and then tested at 1, 24, 48 and 72 hour(s) post treatment.

**Adults**. Twenty-one samples, each containing one *B. pahangi* adult female, were prepared in 198 ul of medium in the same vials described earlier. These 21 samples were grouped into 7 groups of three. These groups included one control group, and three treatment groups for the drugs imatinib mesylate and chloroquine at levels of 100nM, 500nM, and 100uM. Each sample was run pre-treatment, treated, covered, and analyzed at the same time points as described with the L3 treatment.

### Frequency differences

Three groups of 10 *B. pahangi* L3’s were analyzed on the WT immediately upon receipt from FR3. Other L3’s were plated in medium and allowed to slowly degrade while sitting on the bench. After 1 day of monitoring via microscopy, degraded worms showing uncharacteristic and lethargic motion under the scope were then analyzed on the WT. Instead of the normal fluid motion, seen in healthy worms, these worms appeared to flick only one end of their body. The same process was repeated at 48 hours. These data, and data from the fresh L3’s, were then imported into Sigview (www.sigview.com). Fast Fourier Transform (FFT) analysis was done to look at spikes at specific frequencies of motion. By comparing FFT diagrams from blank samples and samples with worms we established that worm motion takes place below 16Hz. Further, visual analysis of many FFT diagrams, specific to the worm type being studied showed some general patterns. Based on these patterns each L3 sample was broken into frequency ranges of 0.1–2Hz, 2–8Hz, and 8–16Hz. This was also carried out for *C. elegans*.

RMS amplitude for each frequency range was summed for each sample and averaged across the population group. Then a ratio was calculated relating the three frequency ranges to each other.

### Statistics

A one-way ANOVA was used, followed by post hoc Tukey’s test to compare the means for each sample group and determine significance of RMS amplitudes for all of the worms run on the WiggleTron [13]. This analysis was done using JMP 11 software (www.jmp.com).

## Results

### Detection of motion

**Fig 2:**
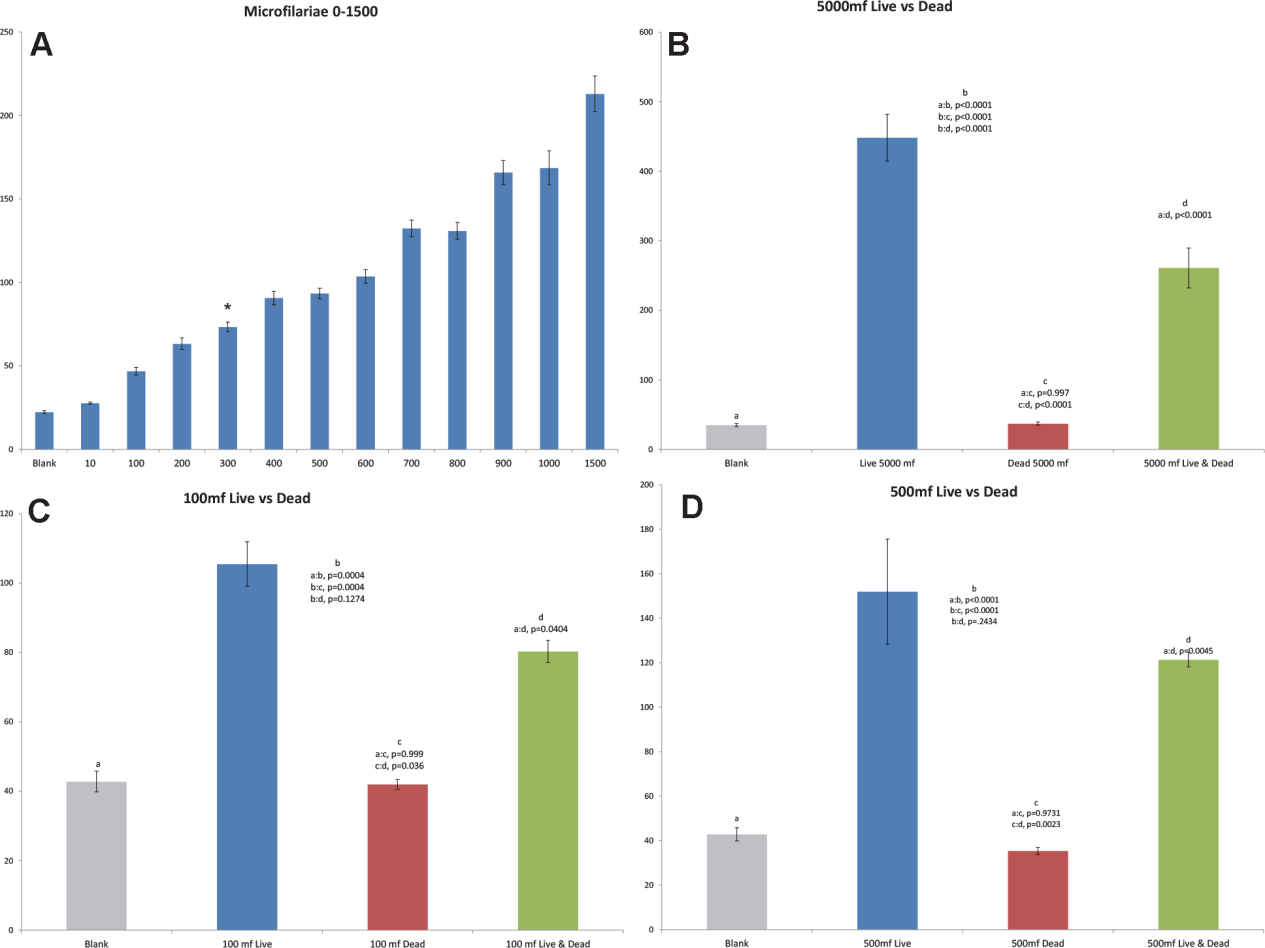
a. Mean returns (y-axis) for *B. pahangi* microfilariae from 0–1500 worms (x-axis) per tube. (*) shows significant difference between 300mf and blank using Tukey’s test (p = 0.0079), **b**. Mean returns for 5000 live microfilariae, 5000 dead microfilariae, and 5000 live & dead. **c**. Mean returns for 100 live microfilariae, 100 dead microfilariae, and 100 live & dead. **d**. 500 live microfilariae, 500 dead microfilariae, and 500 live & dead microfilariae.

**Fig 3:**
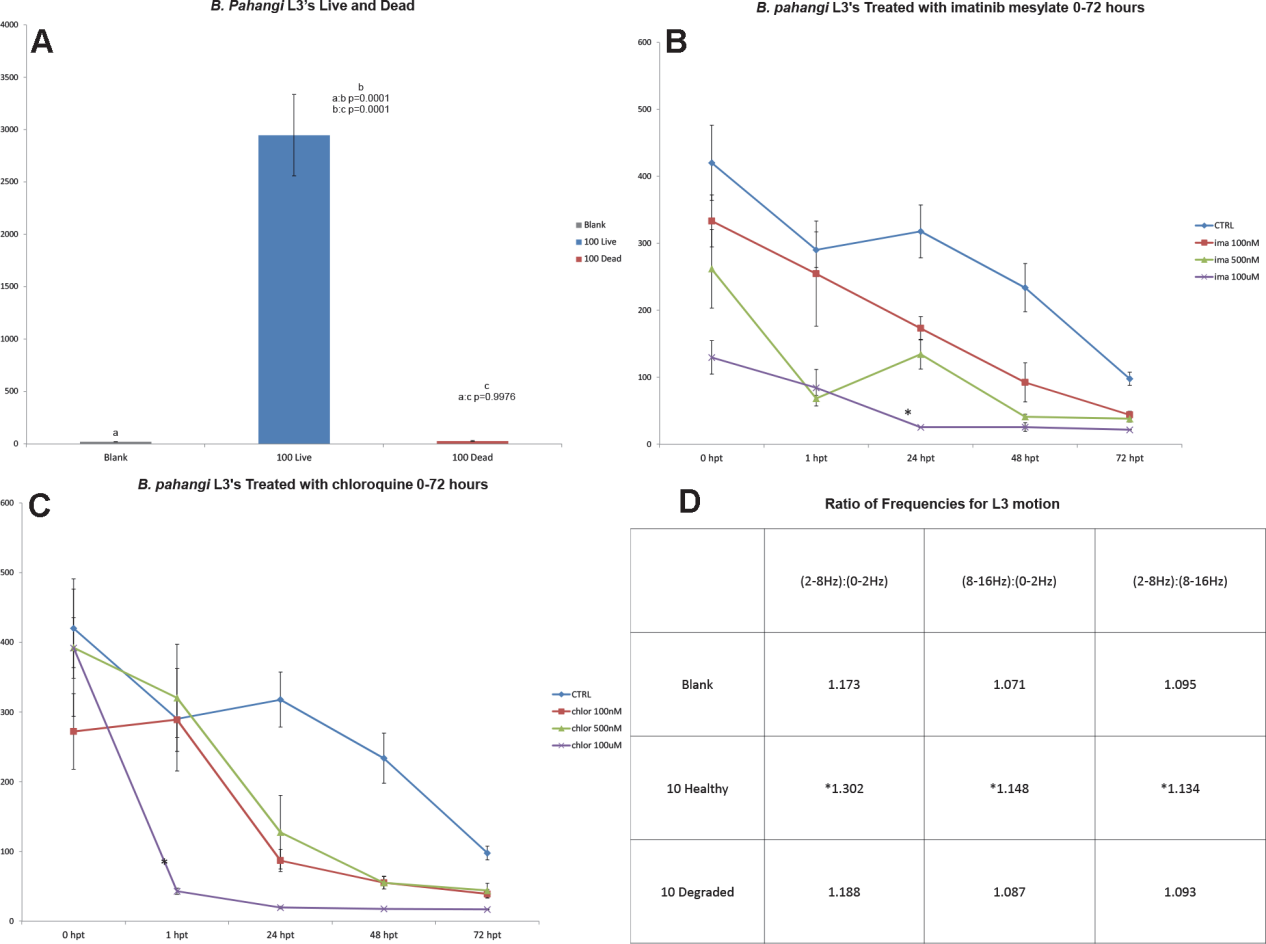
a. Mean RMS amplitudes for blank and 100 live and dead *B. pahangi* L3’s. **b**. Mean RMS amplitudes over time of L3’s treated at different molar concentrations of imatinib mesylate, (*) significant change from pretreatment earlier than control or other groups (p = 0.0153). **c**. Mean RMS amplitudes of L3’s treated at different molar concentrations of chloroquine, (*) significant change from pretreatment earlier than control or other groups (p<0.0001). **d**. Ratios of the maximum amplitudes for the selected frequency ranges of L3 motion, healthy (*) significantly different from blank or degraded (all p<0.0001), blank statistically same as degraded (all p>0.1917)

**Fig 4:**
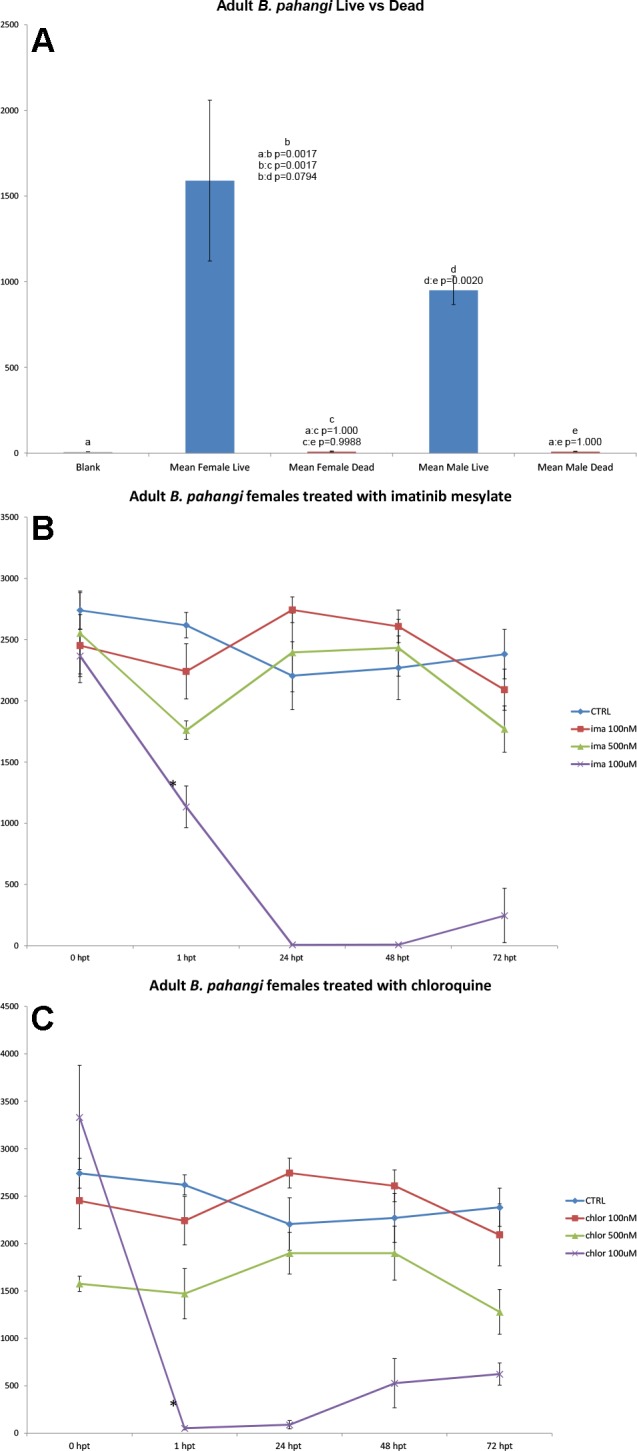
a. Mean RMS amplitudes for live and dead adult male and female *B. pahangi*. **b**. Mean RMS amplitudes for adult females treated with different molar concentrations of imatinib mesylate, (*) significant differences from before drug treatment at 1hpt and on (p<0.0291). **c**. Mean RMS amplitudes for adult females treated with different molar concentrations of chloroquine significant differences from before drug treatment at 1hpt and on (p<0.0346).

**Fig 5:**
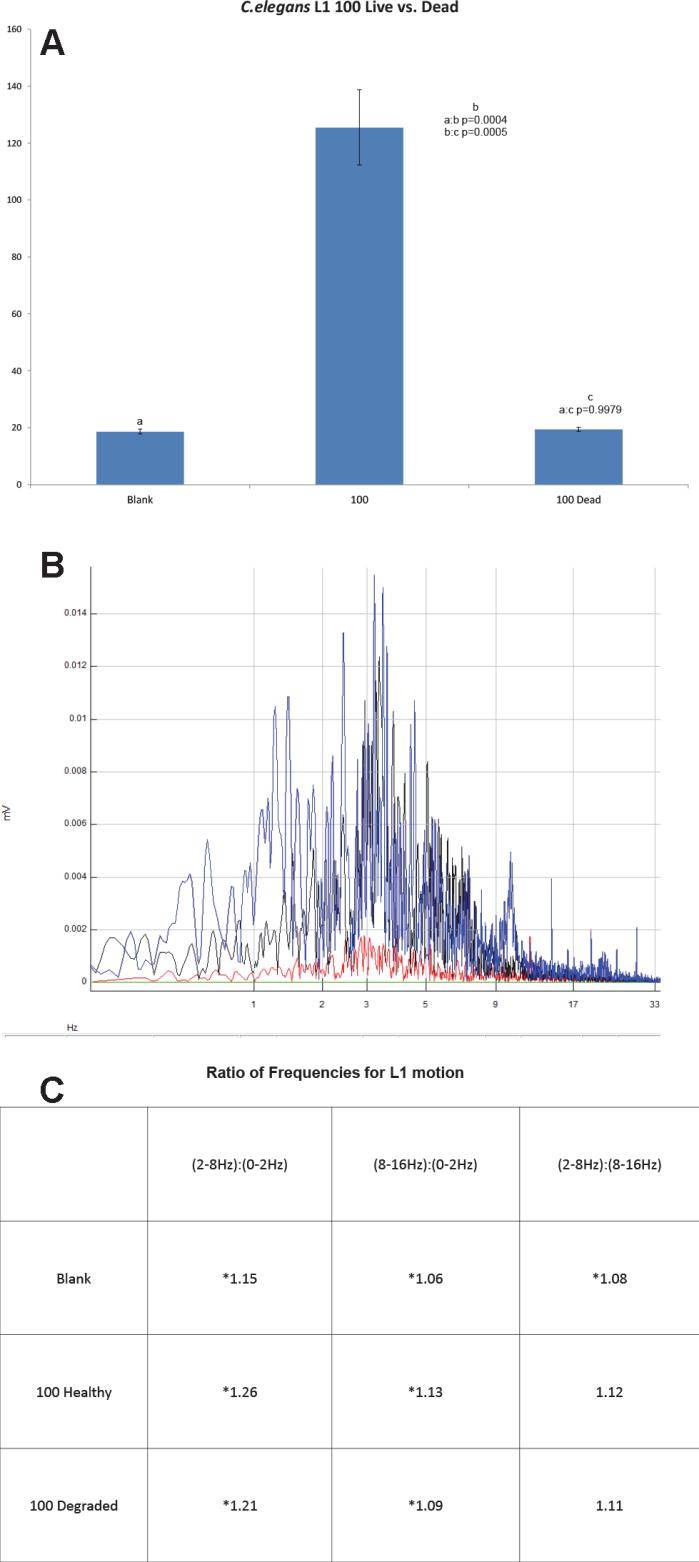
a. Mean RMS amplitudes of *C. elegans* L1 from 0 and 100 worms, live and dead. **b**. FFT of 100 *C. elegans* L1(blue), overlaid with 100 *b. pahangi* (black), and blank (red). **c**. Ratios of selected frequency ranges of *C. elegans* L1 motion, showing significant differences (*) in RMS amplitudes of degraded worms vs blanks (p<0.0001), healthy worms vs blank (p<0.0001), and healthy worms vs degraded in all but the ratio of middle frequency range to high frequency range (p = 0.3095).

Analysis of the data from the WT shows that the device can detect parasite presence and that detection results from parasite motion within the tube. A real-time trace of motion follows a sinusoidal pattern with the majority of the movement, taking place at frequencies less than 20Hz (**Fig. 1B & 1C**). All the worms studied, when healthy, show a periodic motion when viewed under a microscope, bending or twisting around a center point on the worm’s body. This results in a cyclic bending-unbending or twisting-untwisting motion. The detector’s baseline RMS amplitude for no motion is zero. This motion of the worm scatters light, either increasing or decreasing the amount of light received by the photodiodes. This yields an elevated or lessened RMS amplitude based on the direction of motion and nature of the light scattering. Based on the cyclic motion of the worm, a sinusoidal RMS amplitude is detected. Expectedly, due to big physical differences, live worms yield dramatically different RMS amplitudes based on population size, type/species, and larval stage within each tube (**Fig. 1D**). However, despite the difference in their RMS amplitudes, they are all similar in that live worms yield RMS amplitudes that are significantly larger than blank tubes. Results obtained from adults of both sexes, L3’s, microfilariae, and L1’s all show that dead worms are functionally non-existent and give results similar to blank tubes when analyzed within the WT (**Figs. 2B, 2C, 3A, 4A & 5A**). There is however, with parasite numbers well above the normal infection or test levels usually likely to be examined (>500 mf/200ul), a significant impact on RMS amplitude seen. Fig. 2C shows that at 100 or 500 mf/ 200ul there in no effect of adding an equivalent number of dead similar worms.

### Estimation of worm number

Analysis of the data from microfilariae the WT shows that this system can estimate different numbers of worms based on the amplitude of RMS amplitude (motion). For microfilariae there is a positive correlation between amplitude and worm number in samples up to 5,000 worms. With numbers of microfilariae greater than 5,000 the signals resulting from worm motion no longer consistently correlate with increases in worm population. However, two further general characteristics of the WT’s ability to capture motion were seen: firstly the maximum RMS amplitude possible for the present experimental conditions occurs at 17,500 microfilariae, and secondly at numbers higher than 17,500 microfilariae there is a decline in signal with an increase in population. With populations of 0–1500 microfilariae, the increase in RMS amplitude resultant from an increase in population is linear with a sensitivity of 300 worms (p = 0.0079). Analysis of data with *B. pahangi* L3’s, and *C. elegans* L1’s shows an increase in RMS amplitude along with population from 0–100 worms. The *B. pahangi* L3 sensitivity is 5 worms (p = 0.0166), whereas the sensitivity for *C. elegans* L1’s is 50 worms (p = 0.0014). Due to the size of the adult *Brugia* parasites, multiple worms were not run within the same tube for this study. Analysis of over 400 adult worms from the peritoneal cavity of girds shows that adult worm sex can be determined based on amplitude alone through the use of the WiggleTron (p<0.0001).

Using known RMS amplitude values for each population, population as a function of RMS amplitude can be plotted and used to estimate the population size of an unknown sample. Microfilariae were used to investigate the predictive nature as there is a need for a quick, easy field test to confirm and quantify microfilariaemia in endemic populations. Functions generated using results from 0–1,500 microfilariae can estimate within 20% error the population of microfilariae within a tube for numbers of worms less than 200. Multiple tests yielded an error of 16.7% when testing a population of 175 worms. At higher numbers, the non-linear relationship between RMS amplitude and population failed to estimate the population number with an accuracy of +/- 20%, and this makes prediction using the WT unreliable for this situation.

### Analysis of worm health

Analysis of the data from the WT shows that after treatment with prospective anti-filarial drugs, there is a significant difference in the amplitude of the RMS amplitude between treated and control groups. Forty-eight hours post treatment, results from L3’s showed a significant decrease in motion across all treatment groups and the control group (Fig. 3). The treatment groups receiving the highest doses, approximately 100x known lethal dosage (LD100) of each drug, yielded results that were nearly identical to that of dead worms or blank tubes. The changes in worm motion were significant earlier, 1hour post treatment (hpt) for the chloroquine 100uM group, and 24 hpt in the imatinib mesylate 100uM group. The lower treatment levels showed that there was still some motion in the worms, yet their activity level as compared to the control group was greatly reduced. This was confirmed by light microscopy. Analysis of treated adults did not show as drastic results as the L3’s at lower treatment levels, yet the highest treatment groups of each drug showed a significant decrease in activity after treatment. After 72 hours all but the highest treatment levels exhibited some activity (**Fig. 4B & 4C**).

FFT analysis of fresh *B. pahangi* L3’s, and naturally degraded L3’s also shows that the WT can be used to analyze parasite health. There is a peak range in the frequency of motion for these worms between 4Hz and 8Hz (**Fig. 1C**); degraded L3’s lacked activity at this peak range (**Fig. 1C**). This is further shown by comparing the summed motion at different frequency ranges. The healthy L3’s had a significantly different ratio of mid-range frequency of motion compared to high frequency motion or low frequency motion than their microscopically confirmed degraded L3 counterparts (**Fig. 3D**). *C. elegans* showed a peak of motion between the ranges of 3Hz to 8Hz (**Fig. 5B**). When compared to similar sized *B. pahangi* microfilariae, the L1’s showed a different pattern of motion; specifically their peak activity was at higher frequency ranges than the activity of the *Brugia* microfilariae (**Fig. 5B**). When allowed to starve for 48 hours, *C. elegans* L1’s showed significant changes in the pattern of their motion. This is shown by relating the mid-range frequency component of their motion to the low and high range frequency components, then comparing fresh versus starved worms (**Fig. 5C**). The fresh and starved worms had significantly different ratios of mid-range to low and high-range to low (p<0.0001), whereas the ratios of their mid-range motion compared to high was similar (p = 0.3095). Both the fresh and starved worms showed significant differences in signal across all frequency ranges from that of the blank tube (p<0.0001).

## Discussion

Nematodes are organisms of interest to both basic and applied scientists for two major reasons: they are complex entities that can be studied to understand basic biological mechanisms of an early animal organism, and secondly because they cause some of the most devastatingly chronic animal and human diseases in the world. The methodology described in this present communication, the WT, can potentially assist both these communities. By describing the motion of worms in a detailed quantitative manner, with high degree of statistical strength, it is possible to both to record subtle changes in the activity of individual worms and secondly, it can assist in quickly and reliably confirming parasite presence and estimating the numbers of parasites present within the limitations of the machine.

The motion of nematodes has been an area of interest for many years and in many disciplines, including plant parasitology [14], and the biology of the iconic biology research nematode, *C. elegans* [15]. Another important reason to record and understand changes in motion of nematodes is to monitor the effect of external agents, such as anthelminthic drugs, on target parasites. With further testing and improvements to the current WT, it is possible that this technique may be able to detect changes in forms of motion that reflect drug effects on specific organs within the worms; and thus reflect the probable target organs and aid in the search for new anthelmintic agents. Currently the common methods for assessing the effects of potential anthelminthic use a subjective visual assessment of motility, and method that is difficult to standardize between observers. The examples presented here show that depression in motion in the presence of anti-worm compounds (**Figs. 3 & 4**) can be easily visualized and parameters such as time course be recorded.

The issue of the effect of the meniscus is very central to the improvements we have managed to achieve in this current system. The original system of Bennett and Pax [2] utilized the meniscus in the recording. The location of the recording photoreceptor diodes in a position just below the meniscus contributed significantly to the improved sensitivity of this new version of this light-scattering system. Thus in our system the same volume is used in the same type of tube for every experiment and thus the meniscus is positioned in the same place relative to the diodes for each these experiments. This positioning of the photodiodes below the meniscus lessens the effect that the meniscus has on the detection of light changes related to worm motion.

We believe that the system does indeed allow for a “reproducible estimation” of the number of worms present in a small volume, at least within a range that is practically useful for screening the loads of parasites present in solutions, although it is true that this is an approximated figure and not truly a count. We believe nevertheless this does have application in situations where detection of a “cut off” level of parasites is needed, such as screening hyper-infected patients for special treatment. The ability to estimate the numbers of organisms present in small volumes, and to do this with very high numbers of worms present in the solution, i.e. up to approximately 1,000 parasites per ml in the case of filarial microfilariae (**Fig. 2**) could be of interest to epidemiological studies needed for a number of today’s global health programs. Although antigen/antibody tests are the most commonly used currently for parasite disease field programs, there are still occasions where estimation of the number of worms in biological fluid is important and most serological tests are poorly quantitative and sample standard sample tests time consuming or unreliable. In lymphatic filariasis, where there is a major ongoing global program to eliminate the infections and disease, antigen tests can offer false positives and in the case of the ICT antigen test, false negatives [16–18]. The preparation of preparing a blood smear and microscopic examination requires a skilled technician and microscope, as well as potentially exposes the technician to other blood-borne pathogens [15]. This motion based WT system, especially when further miniaturized could be a useful system for field laboratories. This is most urgently needed for screening large numbers of people in endemic areas of loiasis, a filarial worm that when present in high numbers in the blood (> 8,000 mf/ml) can induce fatal reactions following treatment with the standard anti-filarial drug ivermectin [19]; knowing the number of parasites circulating in individuals before treating them with this drug is essential.

The two experiments presented here showing the effects of anthelminthic agents on worm health, using the examples of chloroquine and imatinib (**Fig. 4**), demonstrate that the WT, with its assumed superior objectivity compared to direct observation, is a welcome contribution to the current drug screening efforts. Chloroquine has been shown previously to affect filariae [20], and imatinib (a tyrosine-kinase inhibitor that was originally shown to act on a neoplastic cell enzyme) has been shown to damage the trematode helminth, *Schistosoma mansoni* [21]; our observations here are the first demonstration of anthelminthic effect of imatinib on a filarial nematode.

The ability to analyze and differentiate between healthy and affected nematodes in screening assays could be enhanced using a 96-well plate version of this system. Multiple compounds, at multiple dilutions could be run simultaneously and analyzed for effects on worm movement. Compounds that show promise could be easily identified and investigated further. Refinements in the analysis, through comparison of motion to worm pathology, and video analysis may even allow the machine to identify motion types that are indicative of certain types of damage, narrowing future research into specific mechanisms of damage to the worm.

Although the WT system is currently portable and easy to operate, further development that includes automation of the analytic steps and miniaturization of the equipment could enhance its suitability for use in non-laboratory settings. However, in its present form it already will deliver results that require little more than putting the sample into a tube, introducing the tube to the hand-held collector, and pushing a button on the computer. This system eliminates the need for a specialist technician and allows for multiple analyses and repeated sampling where needed. The ability to function with nematodes of differences sizes, ranging from 300um to 1–2 cm long, brought about by the increased sensitivity to changes in light sensing is likely to be a welcome improvement from older systems. New generations of the instrument focusing on miniaturization would assist in the goal of developing a hand-held version for detection of parasites in settings outside research laboratory, and multi-well plate versions will increase simultaneous sample quantity for more efficient laboratory high throughput screening to enhance the search for new anti-parasite agents.

We have shown here that the WT has the capability of detecting, quantifying, and analyzing the motion of nematodes in a small volume of liquid. This system can be an invaluable tool for field confirmation and quantification of parasite loads, screening a large number of compounds, and confirming drug-induced damage to nematodes; in addition it should greatly enhance further understanding of the biology of movement in nematodes.
